# Social Mobile Approaches to Reducing Weight (SMART) 2.0: protocol of a randomized controlled trial among young adults in university settings

**DOI:** 10.1186/s13063-021-05938-7

**Published:** 2022-01-03

**Authors:** Shadia J. Mansour-Assi, Natalie M. Golaszewski, Victoria Lawhun Costello, David Wing, Hailey Persinger, Aaron Coleman, Leslie Lytle, Britta A. Larsen, Sonia Jain, Nadir Weibel, Cheryl L. Rock, Kevin Patrick, Eric Hekler, Job G. Godino

**Affiliations:** 1 Center for Wireless and Population Health Systems, UC San Diego, La Jolla, CA USA; 2 Herbert Wertheim School of Public Health and Longevity Science, UC San Diego, La Jolla, CA USA; 3 Exercise and Physical Activity Resource Center, UC San Diego, La Jolla, CA USA; 4 Fitabase by Small Steps Labs LLC, San Diego, CA USA; 5 Gillings School of Global Public Health, University of North Carolina, Chapel Hill, NC USA; 6 Department of Computer Science and Engineering, UC San Diego, La Jolla, CA USA; 7 Department of Family Medicine, School of Medicine, UC San Diego, La Jolla, CA USA; 8 Laura Rodriguez Research Institute, Family Health Centers of San Diego, San Diego, CA USA

**Keywords:** Weight loss, Young adults, Wearables, Health coaching, Social media, Digital health, Randomized controlled trial

## Abstract

**Background:**

Excess weight gain in young adulthood is associated with future weight gain and increased risk of chronic disease. Although multimodal, technology-based weight-loss interventions have the potential to promote weight loss among young adults, many interventions have limited personalization, and few have been deployed and evaluated for longer than a year. We aim to assess the effects of a highly personalized, 2-year intervention that uses popular mobile and social technologies to promote weight loss among young adults.

**Methods:**

The Social Mobile Approaches to Reducing Weight (SMART) 2.0 Study is a 24-month parallel-group randomized controlled trial that will include 642 overweight or obese participants, aged 18–35 years, from universities and community colleges in San Diego, CA. All participants receive a wearable activity tracker, connected scale, and corresponding app. Participants randomized to one intervention group receive evidence-based information about weight loss and behavior change techniques via personalized daily text messaging (i.e., SMS/MMS), posts on social media platforms, and online groups. Participants in a second intervention group receive the aforementioned elements in addition to brief, technology-mediated health coaching. Participants in the control group receive a wearable activity tracker, connected scale, and corresponding app alone. The primary outcome is objectively measured weight in kilograms over 24 months. Secondary outcomes include anthropometric measurements; physiological measures; physical activity, diet, sleep, and psychosocial measures; and engagement with intervention modalities. Outcomes are assessed at baseline and 6, 12, 18, and 24 months. Differences between the randomized groups will be analyzed using a mixed model of repeated measures and will be based on the intent-to-treat principle.

**Discussion:**

We hypothesize that both SMART 2.0 intervention groups will significantly improve weight loss compared to the control group, and the group receiving health coaching will experience the greatest improvement. We further hypothesize that differences in secondary outcomes will favor the intervention groups. There is a critical need to advance understanding of the effectiveness of multimodal, technology-based weight-loss interventions that have the potential for long-term effects and widespread dissemination among young adults. Our findings should inform the implementation of low-cost and scalable interventions for weight loss and risk-reducing health behaviors.

**Trial registration:**

ClinicalTrials.govNCT03907462. Registered on April 9, 2019

## Introduction

Overweight and obesity remain major public health concerns in the USA [1, 2]. Recent data from the Centers for Disease Control and Prevention indicate the extent of this problem is great even among young adults. Approximately 62.6% of those 20 to 34 years old are overweight or obese (defined as a body mass index [BMI] ≥ 25 kg/m^2^) [3]. Excess weight gain occurs most rapidly in young adults and is associated with future weight gain [4, 5] and cardiovascular disease risk factors [6].

More than half of young adults in the USA are enrolled in tertiary education [5], and an estimated 40.7% of students are overweight or obese [7]. Engagement in tertiary education represents a period of time when young adults typically undergo the transition from adolescence to young adulthood and often adopt unhealthy weight-related behaviors, such as decreased physical activity [8–10], poor diet quality [8, 9, 11], and poor sleep hygiene [8, 9]. Consequently, many young adults gain weight while in the university or college settings, suggesting a need for evidence-based weight loss interventions that target this population during this transition [12].

One potential strategy is to deploy interventions designed to promote weight loss through healthy changes in physical activity, diet, and sleep [13–15] via mobile and social technologies that are highly pervasive in the USA. For example, approximately 96% of young adults own a smartphone, and 77% of them use it to get information about their health [16, 17]. Furthermore, 21% of adults in the USA regularly wear an activity tracker or smartwatch that monitors health-related outcomes, and device ownership is predicted to rise considerably in the coming years [18]. Social media use among young adults is also ubiquitous, with an estimated 88% using at least one platform regularly and no differences in use by sex, race, or ethnicity [19]. Facebook remains the most popular platform among all adults, and overall engagement is increasing. As many as 70% of adults between the ages of 18 and 29 years old use Facebook daily, and other platforms such as Instagram and Twitter are widely used [19]. Thus, instead of relying on in-person interactions as weight loss interventions have traditionally done [10, 20–23], interventions can utilize mobile- and social media-based modalities to meet young adults in the virtual spaces they frequently inhabit [24, 25]. The flexibility and scalability of this approach may be more acceptable than in-person approaches among this young adult population [26].

Although digital behavior change interventions (DBCI) have shown promising results for weight loss, few studies target young adults and have limited personalization, duration, and modalities [27, 28]. A majority of DBCIs that target weight loss have been conducted exclusively among middle-aged and older adults [27, 28]. Additionally, a minority of interventions include a high level of personalization and multiple behavior change techniques, and few interventions have been implemented for longer than 18 months [27, 28]. The use of several modalities would allow for greater personalization and exposure to intervention content; however, one systematic review found that of the 139 DBCIs included in the review, 60.4% of interventions identified used only one modality, 33.8% two, 5.0% three, and only one used five [28]. Despite these shortcomings, two systematic reviews and meta-analyses have shown that on average, DBCIs can achieve moderate weight loss (− 2.77 kg, 95% CI − 3.54 to − 2.00 kg [27]; − 2.70 95% CI − 3.33 and − 2.08 kg [28]). Thus, there remains a need for long-term, multimodal DBCIs that target weight loss and have the potential for enhanced effect sizes and widespread dissemination among young adults.

### Objectives

The primary objective of the Social Mobile Approaches to Reducing Weight (SMART) 2.0 Study is to determine the effectiveness of our evidence-based, multimodal SMART 2.0 interventions to improve objectively measured weight loss in kilograms over 24 months (96 weeks) compared to a control group. The SMART 2.0 intervention approach is built upon previous DBCIs [29–31], and the study is designed to evaluate the extent to which brief, technology-mediated health coaching might enhance the intervention effects. The secondary objectives are to evaluate the differences between the groups at 6, 12, 18, and 24 months in anthropometric and physiological outcomes, physical activity, diet, sleep, self-esteem, body image, anxiety, and depression. Additional analyses will examine the dose response (i.e., quantified engagement with technological modalities and behavior change techniques) of the intervention, the usability and acceptability of the intervention, potential mediators and moderators of the intervention effects (e.g., contamination), and patterns of change in physical activity, diet, and sleep.

## Methods

### Study design and setting

**Fig. 1:**
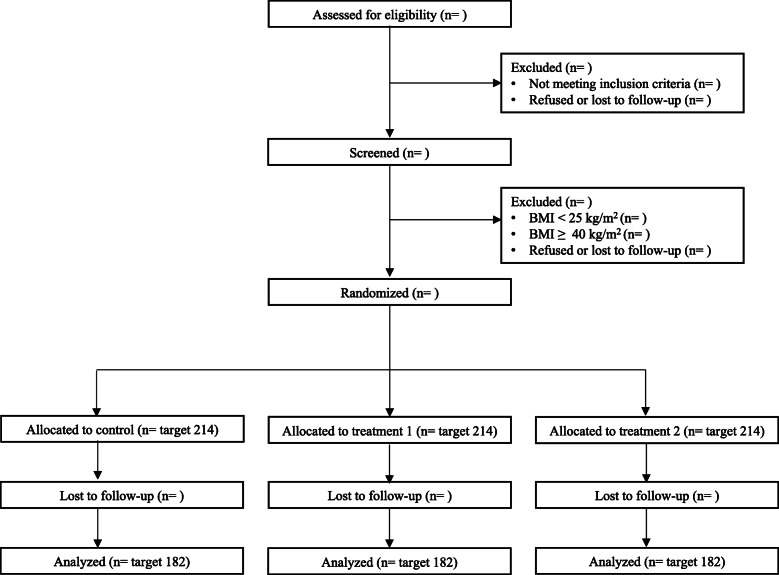
The flow of targeted participants (*N* = 642) through the SMART 2.0 Study

The SMART 2.0 Study is a 24-month (96 weeks) parallel-group randomized controlled trial where 642 overweight and obese young adults, aged 18–35, in San Diego, CA, are randomized to one of three study groups. Participants in each group receive a consumer-level wearable activity tracker and connected scale from Fitbit, which includes access to the Fitbit smartphone and web-based application (app) ecosystem (https://www.fitbit.com/global/us/home). Participants assigned to the control group receive only these technology components. Participants assigned to intervention group 1 (IG1) also receive personalized daily text messages (i.e., SMS/MMS) related to physical activity, diet, sleep, resilience, and weight loss, and access to information about weight loss through study social media pages and a study interventionist-moderated online group. Participants assigned to intervention group 2 (IG2) receive everything that IG1 receives in combination with brief, technology-mediated health coaching. The sponsor and funder, the National Institute of Health, played no part in the study design. They have no role in the collection, management, analysis, and interpretation of the data. They played no part in the writing of the protocol and the decision to submit the protocol for publication. More details on the intervention components are provided below. The study used SPIRIT reporting guidelines [32] and the design and flow of participants are shown in Fig. 1.

### Participants

Young adults aged 18–35 years are recruited from 3 universities and 5 community colleges in San Diego, CA. The eligibility criteria include (1) overweight or obese (25 ≤ BMI < 40 kg/m^2^); (2) available for a 24-month intervention; (3) affiliated with a San Diego University or college as a student, staff, or alumni; (4) willing and able to use social media, a smartphone, text messaging, and Fitbit devices and app; and (5) willing and able to engage in moderate to vigorous physical activity (MVPA). The exclusion criteria include (1) comorbidities of obesity that require a clinical referral or conditions that prohibit compliance with the study protocol, (2) a recent cardiovascular event, (3) currently being treated for malignancy and/or an eating disorder, (4) planning to have weight loss surgery or enroll in a weight loss program, (5) loss of more than 15 pounds within the past 3 months, and (6) pregnancy or planning pregnancy within 24 months.

### Recruitment procedures

Participants are recruited through digital advertisements on campus platforms, targeted social media advertisements, print and digital flyers, email listservs, and campus-wide events. Interested individuals are directed to an online link that includes a description of the study purpose, procedures, risks and benefits, and a secure online screening form. The eligibility criteria are confirmed through telephone screening with the study staff. Participants who meet the eligibility requirements are invited to schedule a baseline appointment at the Exercise and Physical Activity Resource Center at the University of California, San Diego. There, participants are re-screened for the inclusion and exclusion criteria, provided written informed consent, and completed the baseline measurement.

### Retention

To increase enrollment and retention in the study, Fitbit devices and incentives in the form of cash payments and gift cards are provided to all participants. At baseline, each participant receives a wearable activity tracker and connected scale from Fitbit to use throughout the study and keep after study participation is completed. Incentive payments of $20, $25, $25, and $30 are provided at the 6-, 12-, 18-, and 24-month follow-up measurement visits, respectively. Additionally, participants are able to receive the incentive payment for any missed measurement visit(s) if they return for a subsequent visit. For example, if a participant missed their 12-month measurement visit, they are offered $50 ($25 + $25) as compensation for returning for the 18-month visit. Participants affiliated with non-UCSD campuses receive an additional $15 for each measurement visit to compensate for additional travel costs. The appointment scheduling strategy includes automated email reminders sent 3 months prior to each intended measurement visit; continued phone, email, and text message outreach; and flexible scheduling. Lastly, while UCSD in-person research was suspended (March 2020 to August 2020) and is limited (August 2020 to present) to mitigate the spread of COVID-19, remote follow-up measurement visits are offered to participants unable to return to campus. This includes completing self-report surveys online followed by a self-administered weigh-in using their connected scale.

### Randomization, allocation concealment, and blinding

After eligibility and consent are confirmed and baseline measurements are completed, participants are stratified by sex and university/college and then randomized within each stratum at a ratio of 1:1:1 to one of three groups: (1) SMART 2.0 with technology alone (IG1), (2) SMART 2.0 with technology and health coaching (IG2), or (3) a control group receiving only a Fitbit tracker and scale. Each university or college was organized into three strata depending on the nearness of their campus locations. These strata include the following: (1) University of California, San Diego (UCSD); (2) San Diego State University (SDSU), San Diego City Community College District (SDCCD), Grossmont-Cuyamaca Community College District (GCCCD), and Southwestern Community College District (SWCCD); and (3) California State University, San Marcos (CSUSM), Palomar Community College, and MiraCosta Community College. An electronic randomization list was generated using the latest version of the statistical software platform R (version 3.3.2, http://www.r-project.org). The list was securely integrated into the Research Electronic Data Capture (REDCap) tool hosted at UCSD [33, 34], and allocation is concealed from all investigators and staff until the study group is assigned. Only the study manager, health coaches, and research assistants involved in the delivery of the intervention are subsequently made aware of the allocation. All staff that measure participants and investigators that conduct the analyses will remain blinded to the allocation throughout the study.

### Prior research and theoretical foundation

The current study builds on our team of investigators’ previous weight loss interventions [29–31] and addresses newer opportunities for intervention delivery that align with the current state of digital health technology. The ConTxt study, a 12-month personalized text messaging and health coaching intervention, aimed to improve weight among 298 overweight and obese adults, aged 21–60, in San Diego, CA [29]. Adults in the intervention showed a weight loss equal to 3.6%, contrasted against those in the control group who lost 0.6% [29]. The SMART study was part of the Early Adult Reduction of weight through LifestYle (EARLY) trials, a consortium of weight loss studies among young adults [31]. SMART, a 24-month DBCI, used mobile and social technologies (i.e., mobile apps, text messaging, Facebook, emails, a website, and brief health coaching) to improve weight among 404 overweight or obese college students, aged 18–25, from three universities in San Diego, CA. Findings showed significant weight loss (− 1.33 kg) at 6 and 12 months; however, there were no differences at 18 or 24 months [29]. While these studies showed promising findings in improving weight loss, they are not without limitations. Both studies included limited modalities that were not fully integrated into the study design, and participants decreased engagement with technology over time [35]. Findings from exit interviews with 38 participants of the SMART study suggested that an intervention that incorporates popular consumer-level devices and apps, while capitalizing on existing and study-engineered social networks, may be highly engaging to young adults [35–37]. To improve upon its predecessors, the current study uses a fully integrated, highly-tailored system of modalities.

The delivery of theory- and evidence-based content in the SMART 2.0 intervention is flexible and lends itself well to complex and adaptive technology-based interventions that are responsive to an individual’s behavioral progress and ever-changing context [38–41]. We do not have a single overarching theoretical framework, rather the SMART 2.0 intervention content reflects numerous theoretical orientations (e.g., operant conditioning [42], theories of social comparison [43], theories of social support [44], and ecological theory [45]). The use of multiple theories to design the intervention represents a strength of our approach [38–41]. Furthermore, the SMART 2.0 intervention content is mapped directly onto theory-based behavioral change techniques (BCTs). Specifically, the intervention is informed by Abraham and Michie’s taxonomy of 93 distinct BCTs clustered into 16 domains [46]. A meta-analysis of 122 evaluations of interventions that targeted healthy changes in physical activity and diet revealed that the most effective BCTs were self-regulatory and included intention formation, goal setting, self-monitoring, feedback, and goal review [47]. Therefore, contents supporting these are delivered via all modalities, along with content supporting BCTs that target social network mechanisms of influence (e.g., social support, comparison of behavior, and restructuring the social environment). All BCTs included in the intervention were classified prior to delivery (Table 1 describes how these are delivered).

**Table 1:** Description of how intervention content is delivered in the SMART 2.0 Study

Content	Modalities	Description of delivery
Intention formation and goal setting	HC, text messages, online groups	- Health coach facilitates long- and short-term goal setting with the participants during each session. - Data collected by Fitbit and Aria scale prompt tailored weekly goals disseminated via text messages. - Health coach moderates online group discussion so that each group develops and works toward goals.
Self-monitoring	Fitbit activity tracker, connected scale, app, text messages, and social media	- Participants monitor PA, diet, sleep, and weight with on Fitbit devices and in app. - Ongoing self-monitoring is supported by prompts and reminders via text messages and social media.
Feedback	HC, Fitbit activity tracker, connected scale, app, text messages, and online groups	- Health coach provides feedback on participants’ progress on individual goals. - Feedback is provided in real time on Fitbit devices and in app. - Automated text messages containing a summary of individual progress toward reaching tailored weekly goals are sent along with a message of encouragement or positive reinforcement. - Participant posts about progress and/or challenges on social media and in online group and receives feedback from their social network, other participants, and health coach.
Goal review	HC and text messages	- During HC sessions, participants discuss goals and barriers and facilitators for achieving them. - Automated text messages are sent providing feedback on weekly goals and setting a new goal contingent on progress.
Social support and comparison of behavior	HC, text messages, social media, and online groups	- Health coach provides social support during sessions and suggests ways in which participants can seek out support. - Automated text messages include ways in which participants can leverage social support to reach goals. - Participants are connected to other participants and health coach via online groups that are structured to provide positive reinforcement and encouragement.
Restructuring the social environment	Social media, online groups	- Social media and online groups are used to encourage participants to plan PA- and diet-related behaviors together.
Restructuring the physical environment	Social media, online groups	- Information about where to exercise, eat well, and seek mental health resources on campus sent via social media and online groups.

*HC* health coaching, *PA* physical activity

Intervention content is also derived from the strategies for weight management (SWMs), which comprise 35 of the most common evidence-based approaches to achieve weight loss (e.g., reduce portion sizes, avoid processed foods, eliminate sugar-sweetened beverages). The SWMs were successfully integrated into previous studies showing efficacy [48, 49]. Additional intervention content is drawn from comprehensive lifestyle interventions that teach stimulus control, problem solving, time management, and stress management [38, 49].

### Intervention

SMART 2.0 is a multimodal DBCI. Participants in both treatment groups (IG1 and IG2) set a minimum overall weight loss goal of 5% of their baseline weight. Participants are then encouraged to lose 1–2 lb per week until they reach their overall weight loss goal [50]. If a participant reaches a BMI ≤ 25 kg/m^2^, the goal shifts to weight maintenance*.* Through the use of a dynamic text messaging system that is directly integrated with Fitbit data, participants are prescribed weekly physical activity, diet, and sleep goals that begin 1 week after the start of Fitbit data collection. Weekly physical activity goals increase incrementally by 20 min of MVPA building to 225 min (3.75 h) [51]. Weekly dietary goals include achieving a reduced energy intake of at least 500 kcal/day [52], which is monitored through logging dietary consumption via the Fitbit app for 3 consecutive days per week for the first 4 weeks of the intervention, followed by 3 consecutive days per month for the remainder of the study. Weekly sleep goals increase incrementally by 10 min of average nightly sleep building to 420 min (7 h) [53]. Participants are directed to self-monitor their physical activity, diet, and sleep daily and their weight weekly via the Fitbit ecosystem.

### Consumer-level wearable and scale

The wearable activity tracker and connected scale from Fitbit allow for all participants to monitor physical activity, sleep, and diet. Participants receive either the Fitbit Charge 3 or Fitbit Charge 4 as activity trackers and either the Aria 2 scale or Aria Air scale, depending on which was the current consumer-available device at the time of enrollment. The Fitbit Charge 3 and 4 are wrist-worn devices that log objective measurements through its triaxial accelerometer, an optical heart rate monitor, and altimeter. The Aria 2 and Aria Air scales are digital, connected scales that measure weight and BMI. Behavioral and anthropometric trends can then be viewed by the participants in real-time via the Fitbit smartphone or web-based app. Data from the devices is passively and securely streamed to the Fitbit website. It is then retrieved using Fitabase (https://www.fitabase.com), a web-based app developed by Small Step Labs for the simultaneous collection of high-resolution Fitbit data from large numbers of participants and integration with a dynamic text messaging system.

### Text messaging

**Fig. 2:**
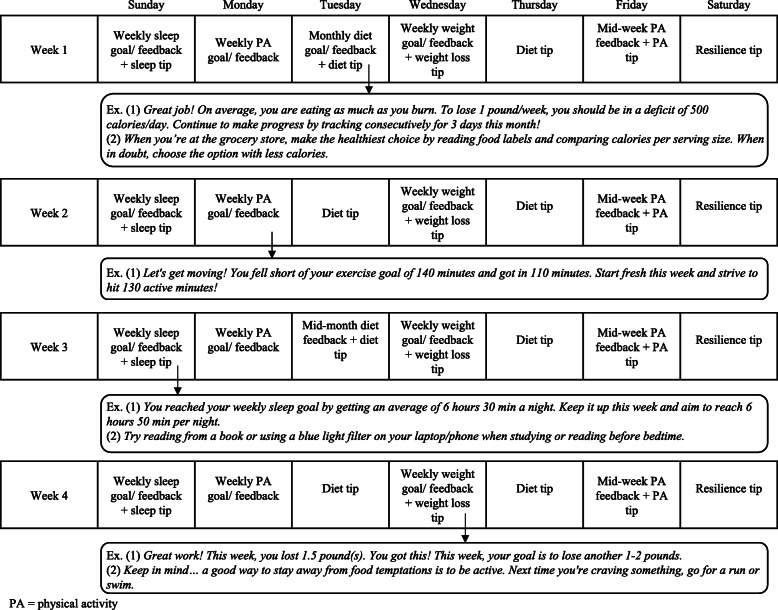
Four-week schedule and examples of text messages delivered in the SMART 2.0 Study

Participants in both treatment groups (IG1 and IG2) receive personalized, tailored goals and feedback, and related behavioral change strategies through daily text messages. These tailored text messages require participants to use all five core self-regulation strategies (i.e., self-monitoring, feedback on performance, behavioral intention formation, goal setting, and goal review) and follow a 4-week format, detailed in Fig. 2. One to two text messages are delivered at a consistent time each day via the Fitabase text message system. Text messages are based on data collected from a participant’s use of the wearable activity tracker and connected scale from Fitbit. Goals and feedback are contingent on a participant’s weight loss and progress in meeting previous weekly physical activity, diet, and sleep behavior goals. For example, after a participant completes their weekly weigh-in, a message will be sent praising the participant for losing 2 pounds and setting a new weekly weight loss goal of 1–2 lb. Additionally, after a participant reaches a BMI < 25 kg/m^2^, goals related to weight, energy intake, and physical activity are automatically adjusted to reflect weight maintenance. For example, if a participant records an average weekly energy intake that is equal to their average weekly energy expenditure, their diet feedback will praise the participant and suggest continued consistent energy intake to maintain their healthy weight.

### Social media and online groups

After randomization, participants in both treatment groups (IG1 and IG2) are placed in groups of 6 to 10 total participants using Facebook messenger (https://www.facebook.com). To do so, each participant connects with a study interventionist as a friend on Facebook, who then adds participants to their respective, private groups on Facebook Messenger. Informational content and resources are shared in the groups and are only accessible to the members of each group a study interventionist. A study interventionist posts content and facilitates group discussion that follows a behavioral weight loss curriculum organized as a series of 24-week (6-month) cycles. This includes knowledge check-ins, shared experiences, and goal setting. A study interventionist monitors all group interactions, responds to inquiries from the participants, and elicits interactions.

A study interventionist also posts general content related to weight loss/management, physical activity, healthy eating, sleep, and resilience to the study’s Facebook, Instagram, and Twitter pages. Participants are required to “like” the SMART 2.0 Facebook page and are encouraged to “follow” the study’s Instagram and Twitter accounts. By “liking” and “following” these respective pages, SMART 2.0 content appears on their individual social media feeds where participants have the option to “like” and “comment” on each post, communicate with other participants through each post, and “save” any content.

### Health coaching

Participants randomized to the IG2 intervention arm receive individual technology-mediated, real-time health coaching that is theory- and evidence-based [46, 54–56]. Health coaching sessions consist of motivational interviewing (MI) [57, 58], participant-determined behavioral goal setting, accountability for health behaviors, health education, and BCTs appropriate for individual coaching [46, 59]. These BCTs include goal setting, action planning, problem solving, feedback on behavior, social support, and instruction on how to perform a behavior [46]. Sessions last approximately 10 min and take place over the phone and/or through Zoom (https://zoom.us), depending on the participant’s preference. Following each session, the health coach sends a session recap via email that includes an outline of what was discussed, a summary of behavioral and weight loss goals, and the date/time of the next session.

In total, participants in IG2 will receive 38 health coaching sessions across 2 years, as shown in Table 2. During year 1 of the intervention, the health coaching curriculum follows the year-long CDC’s Prevent T2 Lifestyle Change Program (DPP). The goals of the DPP include weight loss of 5–7% of participants’ baseline weight in the first 6 months and continued weight loss until participants reach their goal weight [60]. The program emphasizes self-monitoring, self-efficacy, and problem solving and requires weigh-ins at each session and the self-monitoring of diet and physical activity. Health coaches receive this feedback digitally through the Fitbit wearable activity tracker and connected scale. Health coaches cover the DPP curricula during each session and provide participants with the respective DPP handout via email following each session. The DPP session topics have been tailored for the intervention (e.g., replacing in-person group-based activities with related discussion topics) and for the young adult population (e.g., discussing causes and ways to mitigate stress applicable to participants’ lifestyles, such as during final examinations) and is outlined in Table 2. During year 2 of the intervention, participants in IG2 will continue with monthly 10-min sessions with the health coach. Sessions are guided by the participant’s unique behavioral and weight loss goals, rather than predetermined topics used in year 1. Each session also consists of effective health coaching components [54, 56] and BCTs [46].

**Table 2:** The SMART 2.0 Study health coaching year 1 curriculum adapted from the CDC’s National Diabetes Prevention Program (PreventT2)

Session frequency	Session	PreventT2 curriculum topic	SMART 2.0 revision and description
Weekly	1	Introduction to the program	Intro to SMART 2.0: assess motivations and set short- and long-term goals
	2	Track your food	Using the Fitbit app for food tracking: benefits of food tracking, appropriate portion sizes, and reading food labels
	3	Track your activity	Using Fitbit to track PA: benefits of tracking PA and a review of current PA data
	4	Manage stress	Stress and tracking sleep with Fitbit: sources of stress, coping techniques, and the link between stress and sleep and weight
	5	Eat well to prevent T2	Eating well basics: national dietary guidelines
	6	Get active to prevent T2	Getting started getting active: benefits of PA and identify ways to get PA, challenges, and barriers
	7	Burn more calories than you take in	Balancing what you eat and do: review caloric deficit needed for weight loss and tracking caloric balance with the Fitbit
	8	Shop and cook to prevent T2	Meal planning 101: planning and preparing healthy meals
	9	Get more active	Get more active: ways to increase active minutes
	10	Cope with triggers	Navigating triggers to eat unhealthily: ways to cope with and reduce social pressures to eat unhealthily
	11	Find time for fitness	Find time for fitness: challenges and strategies to find time to exercise
	12	Keep your heart healthy	Maintaining a healthy lifestyle: 3-month review of progress and challenges
	13	Take charge of your thoughts	Take charge of your thoughts: mental health and identifying “helpful” versus “harmful” thoughts
	14	Get support	Social support: identify ways to access social support from to support a healthy lifestyle
	15	Eat well away from home	Eating well while eating out and on-campus: challenges and strategies to eating well at restaurants and on campus
	16	Stay motivated to prevent T2	Staying motivated: strategies to stay motivated to exercise, eat well, and sleep
Bi-weekly	17	When weight loss stalls	Weight loss progress and plateaus: strategies to overcome weight loss plateaus and to continue to lose weight
	18	Get enough sleep	Sleep hygiene: benefits of adequate sleep and identify challenges and strategies to getting at least 7 h
	19	Stay active to prevent T2	Staying active: identify challenges and strategies to maintaining consistent PA
	20	Have healthy food you enjoy	Eating healthy on a budget: 6-month review of progress and challenges and strategies to eat healthy on a budget
Monthly	21	More about T2	General health information: identify current challenges and develop strategies to meet goals
	22	Take a fitness break	Quick exercise ideas: brainstorm 10-min exercise ideas and ways to stay active with any schedule
	23	Stay active away from home	Staying on track while on break/vacation: challenges and strategies during school breaks/vacations
	24	More about carbs	More about macronutrients/nutrient-density: macronutrient information and benefits of nutrient-dense foods
	25	Get back on track	Get back on track: review 5-steps to problem solving and strategies for relapse coping and prevention
	26	Prevent T2 for life	Continuing progress in year 2 of SMART 2.0: 1-year review of progress and challenges and create an action plan for year 2

*T2* type 2 diabetes, *PA* physical activity

### Measures

Outcome measures are collected at baseline and 6, 12, 18, and 24 months. The primary outcome, body weight, is objectively measured to the nearest 0.1 kg using a calibrated digital scale (Seca703, Seca GmbH & Co. KG., Hamberg, DE). Secondary outcome measures include anthropometric and physiological outcomes; physical activity, diet, and sleep behaviors; psychological measures; and engagement with intervention modalities. Anthropometric measurements follow standardized procedures and are implemented by trained staff. Height is measured to the nearest 0.1 cm using a stadiometer, BMI is calculated from the height and weight as kg/m^2^, waist and hip circumferences are measured to the nearest 0.1 cm using a stretch-resistant measuring tape, and blood pressure and heart rate are measured with a digital monitor (Critikon Dinamap 8100, GE Healthcare, Chalfont, UK). Body composition and bone density are measured with dual-energy X-ray absorptiometry (DXA). Cardiovascular fitness is assessed through the Tecumseh 3-min step test [61]. Grip strength is measured to the nearest kilogram using a calibrated hydraulic dynamometer (Model Bl55001, FEI, White Plains, NY) [62]. Lower limb and back flexibility are measured using a modified sit and reach test [63].

Physical activity and sleep are objectively measured for 7 days consecutively at the baseline and 12- and 24-month measurement visits using the validated, waist-worn ActiGraph Link (ActiGraph Inc., Pensacola, FL) [64–67] and continuously through Fitbit Charge. Physical activity and sleep are also measured by self-report through the Global Physical Activity Questionnaire (GPAQ) [68], Physical Activity Neighborhood Environment Scale (PANES) [69], and the last 7-day Sedentary and Behavior Questionnaire (SIT-Q-7d) [70]. Social support for engagement in physical activity and sleep are measured using the Physical Activity and Social Support Scale (PASSS) [71], and behavioral perceptions and intentions are measured using items that have been used in prior behavioral health research [72, 73]. Diet is measured using self-report in the Fitbit app and the Diet History Questionnaire III (DHQ-III) [74]. Social support for engagement in a healthy diet is measured using the Social Support for Diet Survey [75], and behavioral perceptions and intentions are measured using items that have been used in prior behavioral health research [76, 77]. Engagement in behaviors typically used to achieve weight loss are measured using the strategies for weight management [48].

Psychological measures include the Center for Epidemiological Studies - Depression (CES-D) scale [78], Spielberger State Trait Anxiety Inventory (STAI) [79], Rosenberg Self-Esteem Scale [80], and Quality of Wellbeing Scale [81]. Among the intervention group participants, objective markers of engagement include usage of the Fitbit wearable activity tracker and connected scale, text messages received, interactions on social media pages and online groups (e.g., liking a post), and amount of health coaching sessions received. At 24 months, usability and acceptability of the intervention are assessed using a Likert scale that asks about the level of satisfaction with the program, each program modality, and overall progress. The enrollment, allocation, and measures are summarized in Table 3.

**Table 3:** Schedule of enrollment, interventions, and measures for the SMART 2.0 Study

	Study period					
	Enrollment	Allocation	Post-allocation			
Time point		0	6	12	18	24
Enrollment						
Eligibility screen	•					
Informed consent	•					
Allocation		•				
Interventions						
SMART 2.0 with technology alone (IG1)		→	→	→	→	→
SMART 2.0 with technology and health coaching (IG2)		→	→	→	→	→
Control group		→	→	→	→	→
Measures						
Anthropometrics and physiological outcomes: weight, height, BMI, waist and hip circumference, blood pressure, and heart rate		•	•	•	•	•
Body composition: DXA		•	•	•	•	•
Bone density: DXA		•		•		•
Cardiovascular fitness, flexibility, and grip strength		•	•	•	•	•
Physical activity: actigraph		•		•		•
Physical activity and sleep: Fitbit activity tracker		→	→	→	→	→
Questionnaires: GPAQ, PANES, SIT-Q-7d, PASSS, DHQ-III, Social Support for Diet, strategies for weight management, CES-D, STAI, Rosenberg Self-Esteem Scale, and Quality of Wellbeing Scale		•	•	•	•	•
Engagement		→	→	→	→	→
Usability and acceptability						•

*BMI* body mass index (kg/m^2^), *DXA* dual-energy X-ray absorptiometry, *GPAQ* Global Physical Activity Questionnaire, *PANES* Physical Activity Neighborhood Environment Scale, *SIT-Q-7d* 7-day Sedentary Behavior Questionnaire, *PASSS* Physical Activity and Social Support Scale, *DHQ-III* Diet History Questionnaire III, *CES-D* Center for Epidemiological Studies Depression Scale, *STAI* State-Trait Anxiety Inventory

### Data management and quality assurance

The principal investigator will be responsible for monitoring data collection, data quality and timeliness, and monitoring participant recruitment, accrual, and retention. All measures are collected and managed using the secure, HIPAA-compliant web-based tool REDCap [33, 34] hosted at UCSD. REDCap provides an intuitive interface for data entry, audit trails for tracking data manipulation and export procedures, automated export procedures for seamless data downloads to common statistical packages, and procedures for importing data from external sources (e.g., all study anthropometric and physiological measures). Data collected will be kept strictly confidential, accessed only by members of the trial team, and stored on a secure database on REDCap. Each participant will be allocated an individual trial identification number. The study is not exceptionally large or long term, and no planned interim analyses for efficacy or futility will be conducted. Therefore, a Data Safety and Monitoring Board will not be appointed. However, adverse events and unanticipated problems involving risk to participants will be monitored weekly throughout the entirety of the randomized controlled trial and reported to the Human Research Protections Programs (HRPP) at UCSD within 10 days. Anticipated adverse events include muscle or bone injury during physical activity, physical discomfort wearing the Fitbit device, and falling, dizziness, nausea, and fatigue during cardiovascular fitness testing. There is no anticipated harm and compensation for trial participation and, thus, no provisions for post-trial care. Additionally, the investigators will protect the health and safety of participants and pursue the research objectives with scientific diligence by monitoring responses to social and behavioral measures, blood pressure, and changes in weight and will inform participants of information relevant to their continued participation. A fidelity of 5% of health coaching sessions will be evaluated by trained research staff using an adapted ASPIRE-VA health coaching fidelity checklist [82]. The trial team, including investigators and research staff, will meet weekly to review trial conduct, and the principal investigator will report to the Study Steering Committee at regular meetings. HRPP at UCSD will review the conduct annually throughout the trial period. The sponsor, funder, and HRPP will be notified of any potential future protocol amendments prior to implementation, and the protocol will be updated in the clinical trials registry. All protocol deviations will be fully documented using a protocol deviation form. Within 6 months after the completion of study analyses, or upon publication of findings, whichever comes first, data will be made available to the scientific research community via a public website and/or data repository. Any data required to support the protocol can be supplied upon request.

### Statistical analysis

Analyses will be conducted using the latest version of the statistical software platform R and will be based on the intention-to-treat principle. All tests of significance will be two-sided and a *p*-value of 0.05 will be considered statistically significant. Summary statistics (e.g., mean, standard deviations, proportions) will be calculated for all variables of interest. Outliers will be assessed, and variables whose distributions depart significantly from normality will be transformed. Appropriate non-parametric alternatives will be considered if parametric assumptions fail.

The primary outcome of the study is the change in objectively measured weight in kilograms, and the SMART 2.0 intervention groups will be compared to the control group using a mixed model of repeated measures (MMRM) [83]. The model will include the change in weight from baseline at each post-baseline visit (i.e., 6, 12, 18, and 24 months) as the dependent variable. Fixed effects will include study group, visit, study group-by-visit interaction, weight at baseline, and any variables determined to be confounders. Visit will be treated as a categorical variable, and an unstructured variance-covariance structure will be used. The results will be reported as point estimates (mean differences between the groups) and interval estimates (95% confidence intervals). An intervention effect will be concluded if the *p*-value for the study group-by-visit interaction contrast in the model at 24 months is statistically significant. Holm’s method will be used to adjust the two *p*-values for multiple comparisons [84]. This approach uses all available data and is robust to data missing at random (MAR) [85, 86]. However, two additional approaches may be employed to examine the influence of missing data on the primary outcome analysis (which takes a likelihood-based approach to estimation but does not directly impute data). First, we will model the probability of missingness as a function of baseline covariates and previous outcomes (using logistic regression). The inverse of the resulting probabilities will serve as propensity scores that will be included in the model of the primary outcome. If data are MAR or the probability of missingness can be fully explained by observable data, this approach produces asymptotically unbiased estimates. Second, in order to allow for the possibility that the MAR assumption may not hold (an assumption that is not empirically testable), we will use pattern mixture models in which the distribution of the primary outcome is assumed to follow a mixture of two distributions: one for those who complete follow-up and another for those who do not. These approaches will allow us to quantify the robustness of the study findings to missing data assumptions.

Secondary outcomes will be analyzed using the MMRM approach outlined above to compare the differences between the SMART 2.0 intervention groups and the control group at 6, 12, 18, and 24 months in anthropometric and physiological outcomes, physical activity, diet, sleep, body image, anxiety, depression, and the frequency and composition of participant’s online communication about weight-related behaviors (all of these measures are continuous). The dose response (i.e., engagement with intervention modalities) of the SMART 2.0 interventions on outcomes at 6, 12, 18, and 24 months will be examined by including engagement variables as independent variables in multiple regression models with the study outcomes as the dependent variable adjusting for covariates. Factors that may mediate or moderate the effect of the SMART 2.0 interventions on study outcomes will be examined. Mediators (e.g., physical activity, diet, social support) will inform how the intervention may have worked to change the outcome, while moderators (e.g., sex, age, social network connectivity, contamination) will illuminate for whom and under what conditions the intervention may have been efficacious. Mediation will be tested via path analysis with regression paths from randomized group to change in the mediator and from change in the mediator to change in the outcome, along with a direct path from the intervention to change in the outcome. Adding interaction terms to the models assessing the intervention effects will test moderation. For all secondary analyses of interest, no adjustments for multiple comparisons will be made and a *p*-value of 0.05 will be considered statistically significant.

### Sample size

In order to ensure that the trial has adequate power to determine the effectiveness of the SMART 2.0 intervention to improve weight loss in kiloograms, we calculated the sample size based on a two-sided, two-sample *t*-test with 80% power at a significance level of 2.5% (a Bonferroni correction to account for two tests). In the SMART study, the standard deviation (SD) of change in weight at 6 and 12 months ranged from 3.87 to 5.97 kg, and we have assumed that the corresponding SD in SMART 2.0 will fall within this range [30]. Furthermore, the smallest statistically significant mean difference in change in weight between the two groups occurred at 12 months and was approximately − 1.33 kg [30]. If we assume an SD of 4.92 and a modest increase in the between-group difference (− 1.60 kg), then we will need 182 subjects per group in order to detect a minimal standardized effect size of 0.33. Thus, we will randomize 642 participants (214 per group accounting for a 15% attrition rate).

## Discussion

There is a need to advance our understanding of the effectiveness of multimodal, technology-based weight-loss interventions that have the potential for long-term effects and widespread dissemination among young adults. By relying on existing mobile and social technology platforms, we are able to meet young adults in the virtual spaces they frequently occupy and deliver evidence-based information about weight loss and behavior change techniques. We hypothesize that both SMART 2.0 intervention groups will significantly improve weight compared to the control group, and the group receiving health coaching will experience the greatest improvement. We further hypothesize that differences in secondary outcomes will favor the intervention groups, with the greatest improvements in the group receiving health coaching.

Findings from the SMART 2.0 study will add to the growing evidence on the effectiveness of DBCIs for weight loss among young adults, and the impact of a long-term intervention that utilizes multiple fully integrated modalities. Importantly, the study will provide insights into the impact of different features of DBCIs, including the potential benefit of technology-mediated health coaching. It will also provide a robust examination of changes in anthropometric and physiological outcomes, weight-loss-related behaviors, and psychosocial outcomes over 2 years. We are also able to explore ways in which different modalities provide opportunities for engagement, usability, and acceptability of intervention content, and potential mediators and moderators of the intervention effects. These findings will inform approaches to promoting regular engagement in physical activity, a healthy diet, and adequate quality sleep. Overall, the findings from this study should inform the implementation of low-cost and scalable interventions for weight loss and risk-reducing health behaviors.

## Trial status

This study is approved by the Human Research Protections Programs at UCSD (protocol #181862, version 1). Recruitment began in April 2019 and was completed in November 2021.

## Data Availability

The datasets analyzed during the current study are available from the corresponding author on reasonable request.

## Abbreviations

SMART: Social Mobile Approaches to Reducing Weight
BMI: Body mass index
DBCI: Digital behavior change intervention
App: Application
IG1: Intervention group 1
IG2: Intervention group 2
MVPA: Moderate to vigorous physical activity
UCSD: University of California, San Diego
SDSU: San Diego State University
SDCCD: San Diego City Community College District
GCCCD: Grossmont-Cuyamaca Community College District
SWCCD: Southwestern Community College District
CSUSM: California State University, San Marcos
REDCap: Research Electronic Data Capture
EARLY: Early Adult Reduction of weight through LifestYle
BCTs: Behavioral change techniques
SWMs: Strategies for weight management
MI: Motivational interviewing
DPP: CDC’s Prevent T2 Lifestyle Change Program
DXA: Dual-energy X-ray absorptiometry
GPAQ: Global Physical Activity Questionnaire
PANES: Physical Activity Neighborhood Environment Scale
SIT-Q-7d: 7-day Sedentary and Behavior Questionnaire
PASSS: Physical Activity and Social Support Scale
DHQ-III: Diet History Questionnaire III
CES-D: Center for Epidemiological Studies Depression scale
STAI: Spielberger State Trait Anxiety Inventory
MMRM: Mixed model of repeated measures
MAR: Missing at random
SD: Standard deviation
HC: Health coaching
PA: Physical activity
